# Bacteriome-Associated Endosymbiotic Bacteria of *Nosodendron* Tree Sap Beetles (Coleoptera: Nosodendridae)

**DOI:** 10.3389/fmicb.2020.588841

**Published:** 2020-10-29

**Authors:** Bin Hirota, Xian-Ying Meng, Takema Fukatsu

**Affiliations:** ^1^National Institute of Advanced Industrial Science and Technology, Tsukuba, Japan; ^2^Department of Biological Sciences, Graduate School of Science, The University of Tokyo, Tokyo, Japan; ^3^Graduate School of Life and Environmental Sciences, University of Tsukuba, Tsukuba, Japan

**Keywords:** symbiosis, bacteria, bacteriocyte, bacteriome, beetle, tree sap, Coleoptera, Nosodendridae

## Abstract

The family Nosodendridae is a small group of tree sap beetles with only 91 described species representing three genera from the world. In 1930s, bacteria-harboring symbiotic organs, called bacteriomes, were briefly described in a European species *Nosodendron fasciculare*. Since then, however, no studies have been conducted on the nosodendrid endosymbiosis for decades. Here we investigated the bacteriomes and the endosymbiotic bacteria of *Nosodendron coenosum* and *Nosodendron asiaticum* using molecular phylogenetic and histological approaches. In adults and larvae, a pair of slender bacteriomes were found along both sides of the midgut. The bacteriomes consisted of large bacteriocytes at the center and flat sheath cells on the surface. Fluorescence *in situ* hybridization detected preferential localization of the endosymbiotic bacteria in the cytoplasm of the bacteriocytes. In reproductive adult females, the endosymbiotic bacteria were also detected at the infection zone in the ovarioles and on the surface of growing oocytes, indicating vertical symbiont transmission via ovarial passage. Transmission electron microscopy unveiled bizarre structural features of the bacteriocytes, whose cytoplasm exhibited degenerate cytology with deformed endosymbiont cells. Molecular phylogenetic analysis revealed that the nosodendrid endosymbionts formed a distinct clade in the Bacteroidetes. The nosodendrid endosymbionts were the most closely related to the bacteriome endosymbionts of bostrichid powderpost beetles and also allied to the bacteriome endosymbionts of silvanid grain beetles, uncovering an unexpected endosymbiont relationship across the unrelated beetle families Nosodendridae, Bostrichidae and Silvanidae. Host-symbiont co-evolution and presumable biological roles of the endosymbiotic bacteria are discussed.

## Introduction

Beetles, characterized by their sclerotized exoskeleton, in particular thick and hard forewings called elytra, constitute the largest insect order Coleoptera with over 400,000 described species in the world, which adapt to and prosper in every available habitat in the terrestrial ecosystem (Grimaldi and Engel, 2005). Reflecting the enormous diversity and underpinning the outstanding ecological success, many beetles are in symbiotic association with a variety of microorganisms in a variety of ways, which are significantly relevant to their physiology, ecology and adaptation (Buchner, 1965; Salem et al., 2015; Biedermann and Vega, 2020; Hosokawa and Fukatsu, 2020; Kaltenpoth and Flórez, 2020).

Some beetles are externally associated with specific microbes, where the ectosymbiotic microbes are either stored within external pouch-like structures called mycangia or attached on the body surface (Grebennikov and Leschen, 2010; Biedermann and Vega, 2020). The well-known examples are bark and ambrosia beetles of the subfamilies Scolytinae and Platypodinae (Curculionidae), which harbor specific ambrosia fungi in their mycangia, inoculate the fungi onto the wall of wooden galleries excavated under the bark, and consume the resulting fungal growth as the nutritional source (Hulcr and Stelinski, 2017). Such mycangium-borne fungal ectosymbioses have been also reported from ship-timber beetles (Lymexilidae), lizard beetles (Erotylidae), stag beetles (Lucanidae), attelabid weevils (Curculionidae), and others (Francke-Grosmann, 1967; Sakurai, 1985; Tanahashi et al., 2010; Toki et al., 2012). In darkling beetles of the subfamily Lagriinae (Tenebrionidae), specific β-proteobacteria of the genus *Burkholderia* are harbored in peculiar cuticular dorsal pouches of larvae and ovipositor-associated transmission organs of adult females extracellularly, representing a unique case of bacterial ectosymbiosis whose function is protection against pathogen infections by producing antibiotics (Flórez et al., 2017; Kaltenpoth and Flórez, 2020). Other beetles are associated with specific gut microbes, which mainly localize to the lumen of intestinal ceca or crypts, and often also invade the gut epithelial cells and migrate to ovipositor-associated transmission organs of adult females. In cigarette and drugstore beetles (Anobiidae), specific gut-inhabiting yeast-like symbiotic fungi provide their hosts with sterols (Pant and Fraenkel, 1954; Noda and Koizumi, 2003), while in leaf beetles of the subfamilies Cassidinae and Donaciinae (Chrysomelidae), specific genome-reduced gut-inhabiting γ-proteobacteria, *Stammera* and *Macropleicola* respectively, supply pectin-degrading enzymes for plant digestion and/or some essential nutrients for their hosts (Salem et al., 2017, 2020; Reis et al., 2020).

On the other hand, some beetles possess specialized cells for hosting endosymbiotic microorganisms, called the bacteriocytes, which often constitute specialized symbiotic organs, called the bacteriomes within their body cavities (Buchner, 1965). The best-studied bacteriome-associated endosymbiotic bacteria in beetles are those of weevils (Curculionidae). Diverse weevils are associated with an ancient γ-proteobacterial lineage *Nardonella* (Lefèvre et al., 2004; Conord et al., 2008; Hosokawa and Fukatsu, 2010; Hosokawa et al., 2015; Anbutsu et al., 2017). *Nardonella* endosymbionts are localized to bacteriomes surrounding the foregut-midgut junction in larvae and ovarial bacteriomes in adult females (Lefèvre et al., 2004; Hosokawa et al., 2015; Anbutsu et al., 2017). Their genomes are streamlined to as small as 0.2 Mb and specialized for provisioning of tyrosine that is massively needed for cuticle sclerotization of the host beetles upon eclosion (Kuriwada et al., 2010; Anbutsu et al., 2017). The host–symbiont relationship entails strict vertical transmission and co-speciation over evolutionary time estimated as longer than 100 million years (Lefèvre et al., 2004; Conord et al., 2008). In some weevil groups, the original *Nardonella* endosymbiont was replaced by different bacteriome-associated bacterial lineages, like γ-proteobacterial *Sodalis* endosymbionts in the *Sitophilus* grain weevils (Heddi et al., 1999; Heddi and Nardon, 2005; Vigneron et al., 2014) and γ-proteobacterial *Curculioniphilus* endosymbionts in the Curculionini acorn weevils (Toju et al., 2010, 2013; Toju and Fukatsu, 2011).

Besides weevils, early histological studies described the presence of bacteriome-associated endosymbiotic bacteria in horned powderpost beetles and grain borers (Bostrichidae) (Mansour, 1934; Buchner, 1954; Huger, 1956), saw-toothed grain beetles (Silvanidae) (Koch, 1931, 1936; Huger, 1956), soft-winged flower beetles (Dasytidae) (Stammer, 1933), false click beetles (Throscidae) (Stammer, 1933), and tree sap beetles (Nosodendridae) (Stammer, 1933). For decades, the microbiological nature of these endosymbiotic bacteria associated with the tiny minor beetles has been elusive, but recent molecular phylogenetic and genomic studies identified the bostrichid endosymbionts as belonging to the Bacteroidetes (Okude et al., 2017; Engl et al., 2018), the silvanid endosymbionts as allied to the bostrichid endosymbionts in the Bacteroidetes (Hirota et al., 2017; Engl et al., 2018), and the dasytid endosymbionts belonging to the γ-Proteobacteria (Weiss and Kaltenpoth, 2016). Meanwhile, the throscid endosymbionts and the nosodendrid endosymbionts are still to be characterized.

The Nosodendridae is a small family of tree sap beetles, containing only 91 described species representing three genera from around the world (Háva, 2019). Both adults and larvae of nosodendrid beetles are found in tree sap or slime flux seeping from tree injuries, but their exact feeding habit and physiology remain unknown (Yoshitomi et al., 2015). Stammer (1933) briefly reported that the European tree sap beetle *Nosodendron fasciculare* possesses bacteria-harboring bacteriomes, although the paper presented neither data nor figures. In his comprehensive book, Buchner (1965) described some more descriptions on the bacteriomes and the endosymbiotic bacteria of *N. fasciculare* by referring to the doctoral thesis of Öhme (1948), but the Öhme’s data have not been published and are thus unavailable. Since then, there has been no report on the endosymbiosis in nosodendrid beetles.

In this study, we investigated two Japanese nosodendrid species, *N. coenosum* and *N. asiaticum*, for their endosymbiotic bacteria and symbiotic organs using molecular phylogenetic and sophisticated histological techniques, thereby reviving the old microscopic observation into the context of modern microbiology.

## Materials and Methods

### Insects

**FIGURE 1:**
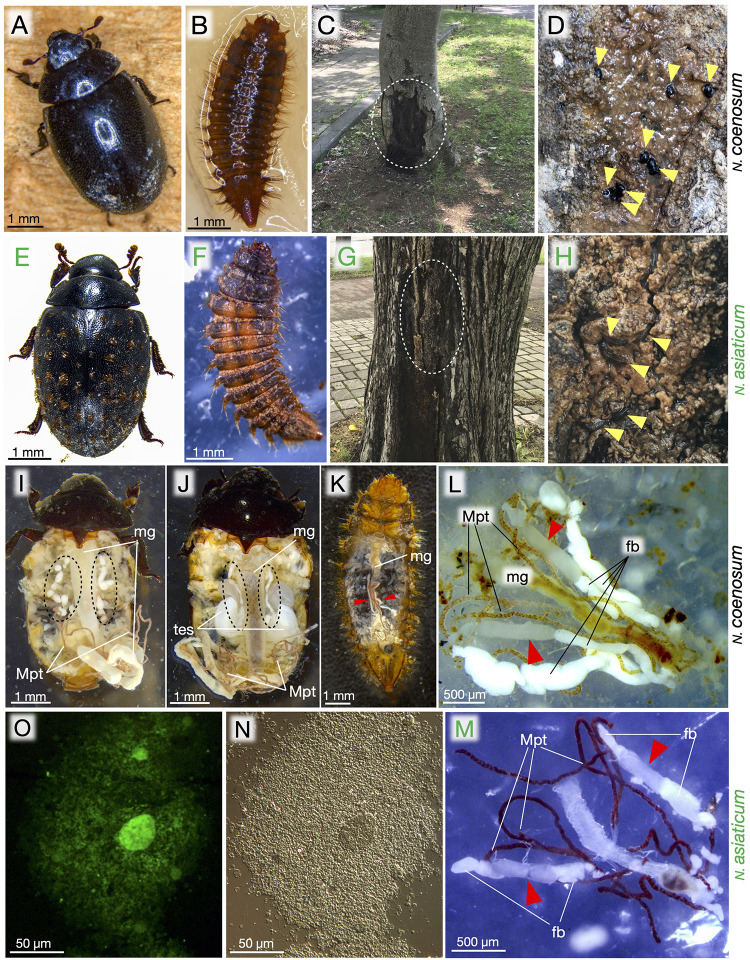
Morphology, ecology, bacteriomes, and endosymbiotic bacteria of *Nosodendron* beetles. **(A–D)** *N. coenosum*. **(E–H)** *N. asiaticum*. **(A,E)** Adults. **(B,F)** Larvae. **(C,D)** Habitat of *N. coenosum*. Tree sap is seeping out of the bark crevices of *Idesia polycarpa*, where adult beetles are seen (yellow arrowheads). **(G,H)** Habitat of *N. asiaticum*. Tree sap is seeping out of the bark crevices of *Ulmus davidiana*, where larvae are found (arrowheads). **(I,J)** Dissected adult female **(I)** and male **(J)** of *N. coenosum*, in which paired sausages-shaped bacteriomes are seen on both sides of midgut (dotted circles). **(K)** A dissected larva of *N. coenosum*, in which a pair of slender bacteriomes are present on both sides of midgut (red arrowheads). **(L,M)** Dissected larval bacteriomes (red arrowheads) of *N. coenosum* **(L)** and *N. asiaticum* **(M)** associated with midgut, fat bodies and Malpighian tubules. **(N,O)** Differential interference microscopic **(N)** and epifluorescence microscopic **(O)** images of endosymbiotic bacteria released from a dissected adult bacteriome of *N. coenosum*. fb, fat body; mg, midgut; Mpt, Malpighian tubule; tes, testis.

Adults and larvae of *N. coenosum* were collected from tree sap of *Idesia polycarpa* or *Aphananthe aspera* (Figures 1A–D, Table 1, Supplementary Table S1). Adults and larvae of *N. asiaticum* were collected from tree sap of *Abies firma* or *Ulmus davidiana* (Figures 1E–H, Table 1, Supplementary Table S1). The insects were kept alive in plastic cases and brought to the laboratory. Some insects were immediately used for histological observations, whereas other insects were either fixed in Carnoy’s solution (ethanol:chloroform:acetic acid = 6:3:1) or preserved in an ultracold freezer at –80°C. By keeping adult insects in plastic cases with paper pieces soaked with tree sap of *I. polycarpa*, a small number of eggs laid on the paper pieces were collected and used for histological examinations.

**TABLE 1:** Nosodendrid beetles examined in this study.

**Insect species**	**Collection locality^a^**	**Collection date^b^**	**Host plant^c^**	**Accession number^d^**
*Nosodendron coenosum*	Tsukuba, Ibaraki	Jun 2019–May 2020	*Idesia polycarpa*	LC567144
*Nosodendron coenosum*	Matsuyama, Ehime	3 Nov, 2019	*Aphananthe aspera*	LC567145
*Nosodendron asiaticum*	Kozagawa, Wakayama	22 Jun, 2019	*Abies firma*	LC567142
*Nosodendron asiaticum*	Sapporo, Hokkaido	9 Aug, 2019	*Ulmus davidiana*	LC567143

*^*a*^All localities are in Japan. ^*b*^All samples were collected by Bin Hirota. ^*c*^Insects were collected from tree sap seeping out of the bark crevices. ^*d*^DDBJ sequence accession number for symbiont 16S rRNA gene sequence.*

### Histological Procedures

For observation of bacteriomes, the insects were placed on silicon-seated Petri dishes (KE-1606, Shin-Etsu Silicone), dissected by tweezers in a phosphate-buffered saline (PBS: 0.8% NaCl, 0.02% KCl, 0.115% Na_2_HPO_4_, 0.02% KH_2_PO_4_) or in 80% ethanol. For observation of fresh endosymbiont cells, a partial piece of the dissected bacteriome was placed on a glass slide with a drop of SYTOX Green solution (1/1,000 dilution), smeared with a coverslip, and observed under an epifluorescence microscope (DM6 B, Leica).

### Fluorescence *in situ* Hybridization (FISH)

FISH targeting bacterial 16S rRNA was conducted as described previously (Koga et al., 2009). Adults, larvae and eggs of the insects were fixed in Carnoy’s solution. The fixed eggs were removed of chorion using fine tweezers. The samples were rehydrated with PBT (PBS containing 0.1% Tween 20) and incubated in a hybridization buffer (20 mM Tris-HCl [pH 8.0], 0.9 M NaCl, 0.01% sodium dodecyl sulfate, 30% formamide, 100 pmol/ml probe) at room temperature overnight. We designed and used a fluorochrome-labeled probe, NosoFlav1263R (5′-AlexaFluor 555-GAT TAG CTT TTA GTC ACC TAA T-3′) that specifically targets the endosymbionts of *N. coenosum* and *N. asiaticum* (Supplementary Figure S1). After washing with PBT three times at room temperature, the samples were placed on glass slides, mounted in 80% glycerol, and observed under a fluorescence dissection microscope (M165FC, Leica), an epifluorescence microscope (Axiophot, Zeiss), and/or a laser scanning confocal microscope (LSM710, Zeiss). To differentiate hybridization signals from autofluorescence and non-specific probe binding, the following control experiments were conducted: no probe control; RNase digestion control; and competitive suppression control with excess unlabeled probe (see Supplementary Figure S2).

### Transmission Electron Microscopy (TEM)

The bacteriomes were dissected from larvae of *N. coenosum* in PBS, prefixed in 2.5% glutaraldehyde in 0.1 M phosphate buffer (pH 7.4) at 4°C overnight, and postfixed with 2% osmium tetroxide in 0.1 M phosphate buffer (pH 7.4) at 4°C for 60 min. After thorough washing and dehydration through a water-ethanol series, the samples were embedded in Epon 812 resin, processed into ultrathin sections (around 80 nm thick) on an ultramicrotome (EM UC7, Leica), mounted on copper meshes, stained with uranyl acetate and lead citrate, and observed under a transmission electron microscope (H-7600, Hitachi).

### DNA Analysis

The dissected tissues were subjected to DNA extraction using QIAamp DNA Mini Kit (Qiagen). A 1.4 kb region of bacterial 16S rRNA gene was amplified by PCR with the primers 10FF (5′-AGT TTG ATC ATG GCT CAG GAT-3′) (Moran et al., 2005) and 1515R (5′-GTA CGG CTA CCT TGT TAC GAC TTA G-3′) (Takiya et al., 2006) under the temperature profile of 94°C for 1 min followed by 30 cycles of 98°C for 10 s, 50°C for 15 s, and 72°C for 2 min. After checking successful PCR amplification by electrophoresis on 1.5% agarose gels, the PCR products were purified using exonuclease I (New England Biolabs) and shrimp alkaline phosphatase (Takara) at 37°C for 15 min followed by 80°C for 15 min, and sequenced as described previously (Fukatsu and Nikoh, 1998).

### Molecular Phylogenetic Analyses

The 16S rRNA gene sequences were subjected to multiple alignment using MUSCLE (Edgar, 2004) implemented in MEGA X (Kumar et al., 2018). The aligned sequence data were inspected and corrected manually, and subjected to the following molecular phylogenetic analyses: neighbor-joining analysis using MEGA X with 1,000 bootstrap replicates; maximum-likelihood analysis using MEGA X with 1,000 bootstrap replicates; and Bayesian analysis using MrBayes v3.2.7 (Ronquist et al., 2012). The best-fit substitution models for the aligned sequences were evaluated by MEGA X for the maximum-likelihood method and by Kakusan v4 (Tanabe, 2011) for the Bayesian method, which selected the generalized time-reversible gamma model for both the methods.

## Results

### Localization and Shape of Bacteriomes

When adult females of *N. coenosum* were dissected in 80% ethanol, a pair of linked sausages-shaped bacteriomes were identified beneath the ovaries in association with the Malpighian tubules and the midgut, where each bacteriome consisted of round- or bean-shaped bacteriome lobes serially connected to each other via tracheae (Figure 1I). In adult males of *N. coenosum*, a pair of linked sausages-shaped bacteriomes, which looked slightly thinner than those in adult females, were found on lateral sides of the midgut and extending to dorsal side of the testes (Figure 1J). In larvae of *N. coenosum*, a pair of slender bacteriomes were present along the ventro-lateral sides of the midgut in association with the Malpighian tubules and the fat bodies (Figures 1K,L). Similar morphological features of the bacteriomes were observed in larvae of *N. asiaticum* (Figure 1M). Smearing of a piece of bacteriome lobe on a glass slide released a dense population of bacterial cells (Figures 1N,O).

### Bacterial 16S rRNA Gene Sequences From Bacteriomes

We dissected the bacteriomes from four larvae of *N. coenosum*, two from Tsukuba population and two from Matsuyama population (see Table 1), and the dissected bacteriomes were subjected to DNA extraction, PCR amplification and sequencing of bacterial 16S rRNA gene. From all the insects, nearly identical 1,439 bp nucleotide sequences were identified, with three bases difference between the populations, which showed extremely adenine-thymine (AT) biased nucleotide compositions at 61.7% (888/1,439) – 61.9% (891/1,439). We also dissected the bacteriomes from four larvae of *N. asiaticum*, two from Sapporo population and two from Kozagawa population (see Table 1), which yielded completely identical 1,448 bp nucleotide sequences from all the insects with AT-biased nucleotide composition at 64.3% (931/1,448). The bacterial sequence similarity between *N. coenosum* and *N. asiaticum* was 83.1% (1,209/1,454). BLASTN searches of the DNA databases using the sequences as queries retrieved 16S rRNA gene sequences of endosymbiotic bacteria of bostrichid beetles as the top hits (e.g., GenBank accession numbers MF183964 and MF183966).

### Phylogenetic Placement of Endosymbiotic Bacteria

**FIGURE 2:**
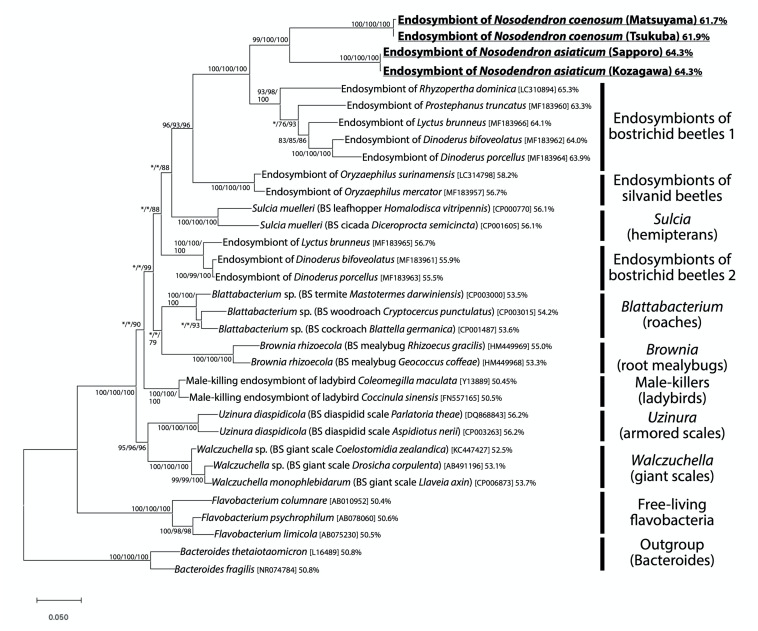
Phylogenic placement of the endosymbiotic bacteria of *Nosodendron* beetles based on 16S rRNA gene sequences. A maximum-likelihood phylogeny inferred from 1,178 aligned nucleotide sites is shown. Statistical support probabilities are shown on each node in the order of neighbor-joining/maximum- likelihood/Bayesian methods, where values less than 70% are indicated by asterisks. For each bacterial sequence, host-related information in parentheses, accession number in brackets, and AT content in percentage are shown. “BS” indicates bacteriocyte-associated endosymbiont. The explanation of each major clade is depicted on the right side.

Figure 2 shows the phylogenetic placement of the bacterial 16S rRNA gene sequences obtained from the bacteriomes of *N. coenosum* and *N. asiaticum*. The bacterial sequences from the nosodendrid beetles formed a highly supported clade in the phylum Bacteroidetes, which was allied to the endosymbiont clades associated with other insect groups, such as endosymbionts of bostrichid powderpost beetles (Okude et al., 2017; Engl et al., 2018), endosymbionts of silvanid grain beetles (Hirota et al., 2017; Engl et al., 2018), *Sulcia* endosymbionts of diverse hemipterans (Moran et al., 2005; Bennett and Moran, 2015), *Walczuchella* endosymbionts of giant scales (Matsuura et al., 2009; Rosas-Pérez et al., 2014), *Uzinura* endosymbionts of armored scales (Gruwell et al., 2007; Andersen et al., 2010), *Blattabacterium* endosymbionts of cockroaches and primitive termite (Bandi et al., 1995), *Brownia* endosymbionts of root mealybugs (Gruwell et al., 2010), etc.

### Bacteriome Localization of Endosymbiotic Bacteria

**FIGURE 3:**
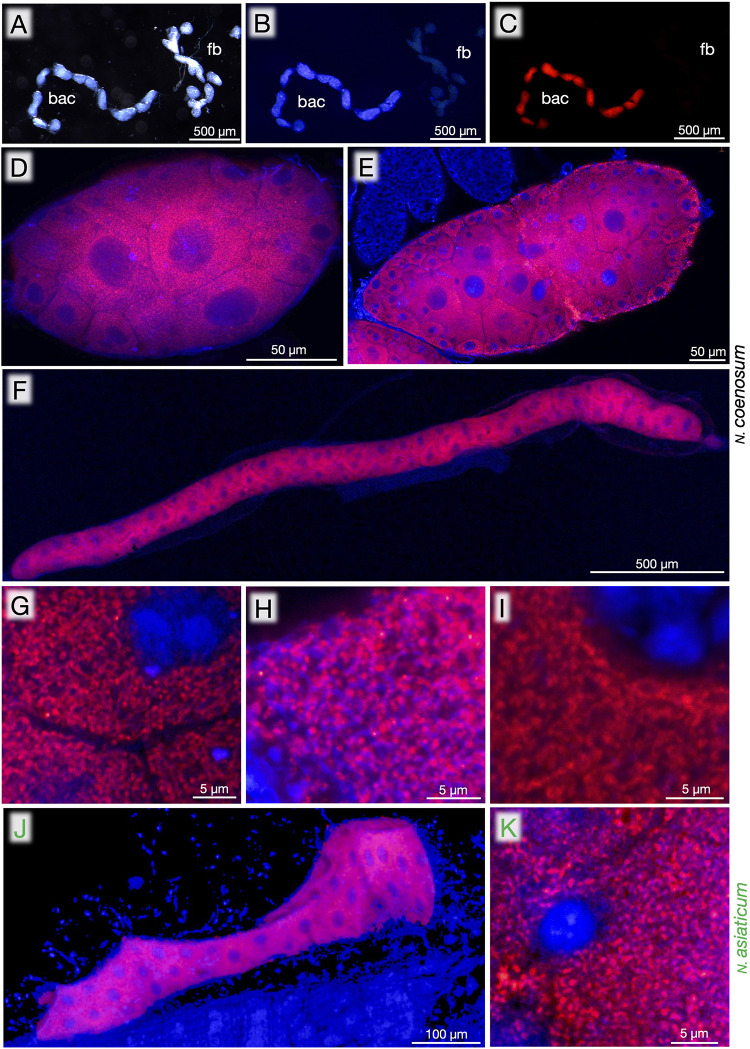
Bacteriomes, bacteriocytes and endosymbiotic bacteria of *Nosodendron* beetles visualized by FISH. **(A–C)** Dissected bacteriomes (bac) and fat bodies (fb) of adult male of *N. coenosum*. **(A)** Light microscopic image. **(B)** Epifluorescence microscopic image in which DNA is visualized in blue. **(C)** Epifluorescence microscopic image in which the endosymbiotic bacteria are visualized in red. **(D–I)** Laser confocal microscopic FISH images of bacteriomes, bacteriocytes and endosymbiotic bacteria of *N. coenosum*. **(D,G)** Adult males. **(E,H)** Adult females. **(F,I)** Larvae. **(D–F)** Images of a whole bacteriome lobe. **(G–I)** Magnified images of the endosymbiotic bacteria in the host cytoplasm. **(J,K)** Laser confocal microscopic FISH images of the bacteriome **(J)** and the endosymbiotic bacteria **(K)** of larval *N. asiaticum.* Red signals represent the endosymbiotic bacteria visualized by FISH targeting 16S rRNA, whereas blue signals show DNA visualized by DAPI staining.

In order to identify *in vivo* localization of the endosymbiotic bacteria, dissected organs of *N. coenosum* were subjected to whole-mount FISH using a fluorochrome-labeled oligonucleotide probe that specifically targets 16S rRNA of the nosodendrid endosymbionts. In adults of *N. coenosum*, bacteriome lobes and fat bodies looked morphologically similar (Figure 3A), but DNA staining and FISH clearly identified specific endosymbiont localization to the bacteriome lobes (Figures 3B,C). In adult males, adult females and larvae of *N. coenosum*, their bacteriomes were densely populated by the endosymbiotic bacteria in the cytoplasm (Figures 3D–I). In the adult bacteriome lobes, the central bacteriocytes tended to be larger in size than the peripheral bacteriocytes, where the central bacteriocytes were often multi-nucleated with a large central nucleus and several small peripheral nuclei (Figures 3D,E). In the larval slender bacteriomes, the bacteriocytes were smaller and uniform (Figure 3F). Similar cytology of the bacteriocytes and localization of the endosymbiotic bacteria were observed in the larval bacteriomes of *N. asiaticum* (Figures 3J,K).

### Localization of Endosymbiotic Bacteria During Oogenesis and Embryogenesis

**FIGURE 4:**
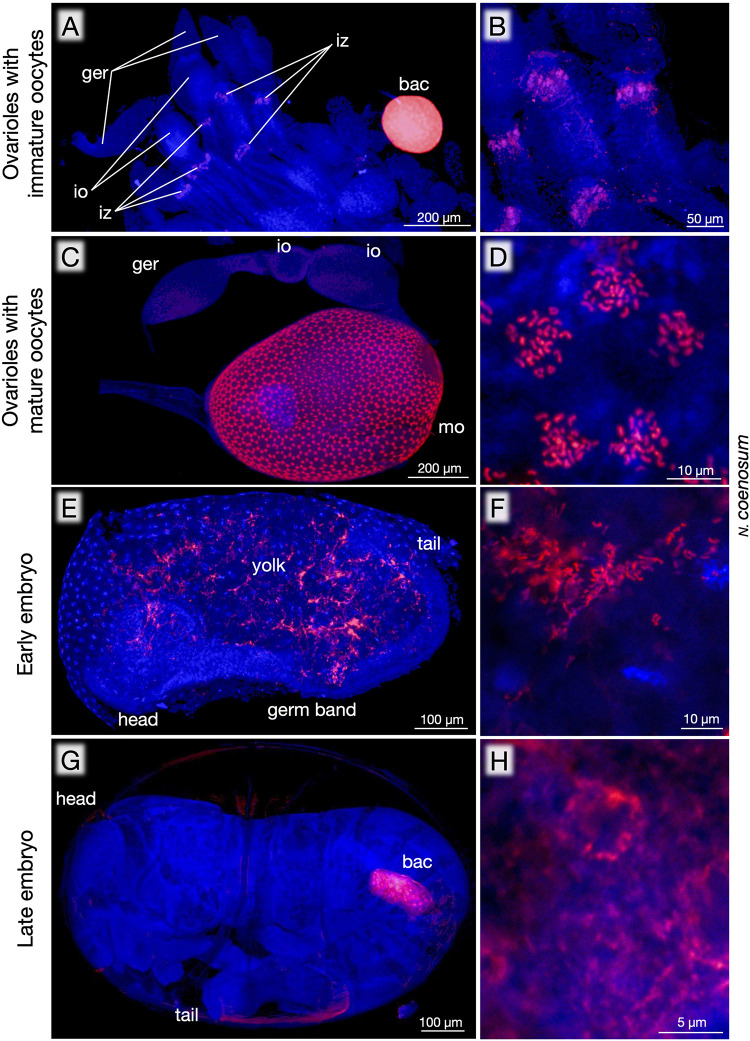
Localization of endosymbiotic bacteria during oogenesis and embryogenesis of *N. coenosum*. **(A)** In ovarioles with immature oocytes, an infection zone for presumable entry region for the endosymbiotic bacteria is detected at the posterior side of the immature oocytes. Note the very strong FISH signal in the bacteriome, highlighting preferential localization of the endosymbiotic bacteria to the symbiotic organ. **(B)** Magnified image of the infection zones. **(C)** In ovarioles with growing oocytes, the egg surface exhibits dense and radial distribution patterns of the endosymbiotic bacteria. **(D)** Magnified image of the endosymbiont cells constituting a radial unit. **(E)** An early embryo, in which the endosymbiont cells are found in the yolk region outside the germ band. **(F)** Magnified image of the endosymbiont cells in the early embryo. **(G)** Side view of a late embryo, in which one of paired primordial bacteriomes is seen within the abdomen. **(H)** Magnified image of the endosymbiont cells in the late embryo. Red signals represent the endosymbiotic bacteria visualized by FISH targeting 16S rRNA, whereas blue signals show DNA visualized by DAPI staining. bac, bacteriome; ger, germarium; io, immature oocyte; iz, infection zone; mo, mature oocyte. Also see Supplementary Figure S2 for negative controls corresponding to **(C)**.

Besides the bacteriomes, whole-mount FISH detected the endosymbiont signals in the ovaries of adult females of *N. coenosum*. In reproductively immature adult females, an infection zone was observed in the middle of each ovariole, where the endosymbiotic bacteria infect presumably for establishing vertical transmission (Figures 4A,B), as observed in *Nysius* seed bugs and other insects (Buchner, 1965; Matsuura et al., 2012). While the infection zone is located at the anterior side of immature oocytes in *Nysius* spp. (Matsuura et al., 2012), the infection zone was at the posterior side of immature oocytes in *N. coenosum* (Figures 4A,B), plausibly reflecting a different transmission passage. In reproductively active adult females, conspicuous distribution patterns of the endosymbiotic bacteria were seen on the surface of growing oocytes in the ovarioles: radially arranged bacteria-infected patches were densely distributed all over the egg surface (Figures 4C,D). What cellular or subcellular structures on the developing oocytes correspond to the conspicuous bacterial localization patterns is of great interest but currently elusive, which deserves future studies. By keeping adult insects in the laboratory, we managed to collect a small number of viable eggs (see Supplementary Table S1), which were analyzed by whole-mount FISH to obtain some snapshot images of the endosymbiont infection process during the embryogenesis of *N. coenosum*. In early embryos, the endosymbiont cells were found in the yolk region outside the germband (Figures 4E,F). In late embryos, the endosymbiont cells were localized to paired primordial bacteriomes in the abdomen (Figures 4G,H).

### Fine Structure of Bacteriomes and Endosymbiotic Bacteria

**FIGURE 5:**
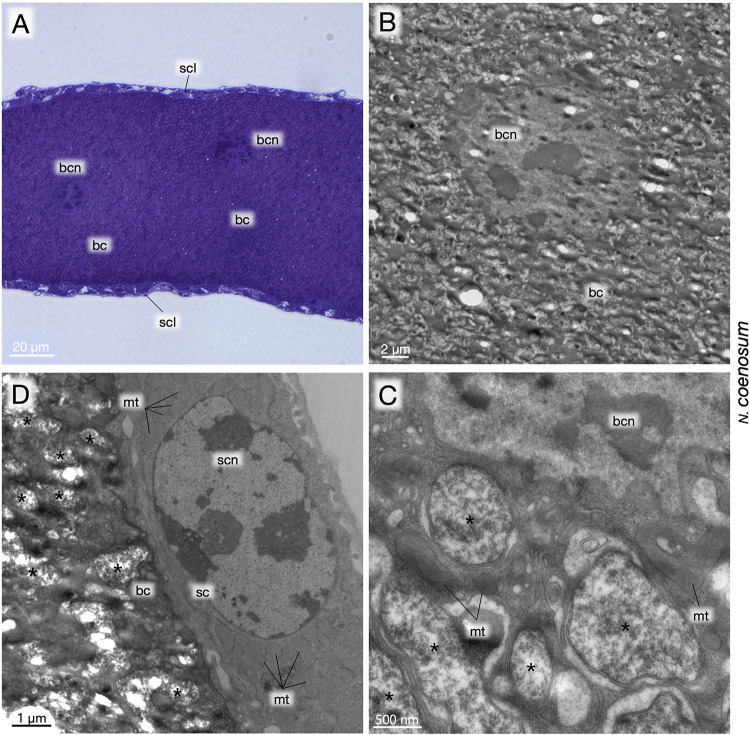
Fine structure of larval bacteriomes, bacteriocytes and endosymbiotic bacteria of *N. coenosum*. **(A)** Semi-ultrathin section of a bacteriome stained with toluidine blue. Voluminous bacteriocytes full of endosymbiotic bacteria are surrounded by a thin sheath cell layer on the surface. **(B)** TEM image of a bacteriocyte, whose cytoplasm exhibits bizarre cytological features such as degenerate cytology, vacuolization, deformed endosymbiont cells, etc. **(C)** Magnified TEM image of the cytoplasm-nucleus interface of a bacteriocyte. Pleomorphic symbiont cells are found in the cytoplasm with mitochondria and accumulated membranous elements. **(D)** Magnified TEM image of the periphery of a bacteriome adjacent to a sheath cell. Note that, while the bacteriocyte exhibits degenerative cytological features, the sheath cell looks normal, confirming that the degenerative cytology of the bacteriocyte is not due to histological artifacts. Asterisks indicate endosymbiont cells. bc, bacteriocyte; bcn, bacteriocyte nucleus; mt, mitochondrion; sc, sheath cell; scl, sheath cell layer; scn, sheath cell nucleus.

Finally, the larval bacteriomes of *N. coenosum* were embedded in epoxy resin and subjected to transmission electron microscopy (TEM). Light microscopic observations of semi-ultrathin sections revealed the cytological configuration of the bacteriome, in which voluminous bacteriocytes full of the endosymbiotic bacteria were surrounded by a thin sheath cell layer on the surface of the bacteriome (Figure 5A). TEM observations of ultrathin sections uncovered bizarre structural features of the bacteriocytes, whose cytoplasm exhibited degenerate cytology, vacuolization, and irregularly shaped endosymbiont cells (Figures 5B,C). At a glance, we suspected that these degenerative traits might be due to experimental fixation artifacts. However, on the same ultrathin sections, the sheath cells exhibited normal fine structures (Figure 5D), indicating that the degenerative cytological traits of the bacteriocytes and the endosymbiotic bacteria are probably the norm.

## Discussion

Since the early brief observational report on the European tree sap beetle *N. fasciculare* (Stammer, 1933), microbiological, histological and biological aspects of the endosymbiosis in the small and inconspicuous nosodendrid beetles have been totally ignored for decades. Here we present microbiological characterization of the endosymbiotic bacteria and histological investigation of the symbiotic organs of two Japanese species, *N. coenosum* and *N. asiaticum*, thereby broadening our knowledge on the diversity of beetle-microbe endosymbiotic associations. This study provides the first microbiological identification of bacteriome-associated endosymbiotic bacteria from the beetle family Nosodendridae.

In all individuals, populations and species examined in this study, the bacteriomes and the associated endosymbiotic bacteria are identified (Figures 1, 3). The endosymbiotic bacteria are localized to specific paired bacteriomes in both larvae and adults, and vertically transmitted to the next generation via ovarial passage (Figures 3, 4). The endosymbiotic bacteria form a distinct and well-supported clade in the Bacteroidetes (Figure 2). Meanwhile, the endosymbiotic bacteria are substantially identical within each *Nosodendron* species whereas they are considerably divergent genetically (83.1% 16S rRNA gene sequence similarity) between *N. coenosum* and *N. asiaticum* (Figure 2). These results seem to favor the hypothesis that the nosodendrid beetles and the bacteriome-associated endosymbiotic bacteria are in intimate association that has been maintained by stable vertical transmission over evolutionary time, although more nosodendrid species should be inspected to confirm this idea.

The degenerative features of the endosymbiont cells (Figure 5) and the extremely AT-biased nucleotide compositions (61.7–64.3%) of the endosymbiont genes (Figure 2) suggest the possibility of drastic genome reduction in the nosodendrid endosymbionts, considering that such cytological and genetic features are generally found among genome-reduced endosymbiotic bacteria (McCutcheon and Moran, 2012). Note that the AT contents of 62–64% are remarkably higher than those of allied genome-reduced endosymbionts of other insects: ∼56% in *Sulcia* of hemipterans with 0.19–0.29 Mb genome (McCutcheon and Moran, 2007; Bennett and Moran, 2013; Koga and Moran, 2014); ∼54% in *Blattabacterium* of cockroaches with 0.59–0.64 Mb genome (López-Sánchez et al., 2009; Sabree et al., 2009, 2012); ∼56% in *Uzinura* of armored scale insects with 0.26 Mb genome (Sabree et al., 2013); ∼53% in *Walczuchella* of giant scale insects with 0.31 Mb genome (Rosas-Pérez et al., 2014); etc. (see Figure 2). Genome sequencing and analysis of the endosymbiont of *N. coenosum* are currently in progress. On the other hand, the possibility should be also taken into account that the degenerative features of the bacteriocytes might be relevant to molting and/or metamorphosis processes of the holometabolous insect host (Hammer and Moran, 2019; Maire et al., 2020).

Phylogenetically, the nosodendrid endosymbiont clade is sister to the bostrichid endosymbiont clade, and they together form a clade allied to the silvanid endosymbiont clade, all of which are highly supported statistically (Figure 2). These patterns highlight the phylogenetic affinity of the bacteriome-associated endosymbiotic bacteria across the beetle families Nosodendridae, Bostrichidae, and Silvanidae. It seems relevant that, like the nosodendrid endosymbionts, the bostrichid endosymbionts and the silvanid endosymbionts exhibit degenerate cytology (rosette-shaped in bostrichids and extremely elongated in silvanids) and AT-biased nucleotide compositions (63–65% in bostrichids and 57–58% in silvanids) (Hirota et al., 2017; Okude et al., 2017; Engl et al., 2018). On the other hand, the endosymbiont relatedness does not agree with the systematics of the host beetles: the family Nosodendridae is placed within the superfamily Nosodendroidea, the family Silvanidae belongs to the superfamily Cucujoidea, the family Bostrichidae belongs to the superfamily Bostrichoidea, and the Nosodendroidea is phylogenetically not as close as the relationship between the Cucujoidea and the Bostrichoidea (Zhang et al., 2018; McKenna et al., 2019). These phylogenetic patterns suggest either of the evolutionary scenarios: (i) independent endosymbiont acquisitions occurred in the Nosodendridae, the Silvanidae and the Bostrichidae from the common or closely related ancestral bacteria belonging to the Bacteroidetes, or (ii) lateral endosymbiont transfers occurred between the Nosodendridae and the Bostrichidae.

Here we observed peculiar localization patterns and presumable vertical transmission routes of the endosymbiotic bacteria during oogenesis and embryogenesis of *N. coenosum* (Figure 4). However, our observations in this study are fragmentary due to limited availability of viable eggs of *N. coenosum*. For obtaining the whole picture of the transmission processes and mechanisms of the endosymbiotic bacteria, we are currently trying to establish a laboratory rearing system of *N. coenosum*.

Biological roles of the bacteriome-associated endosymbionts of the nosodendrid beetles are currently elusive. The majority of hemipteran insects directly suck plant sap from phloem or xylem tubes using a needle-like mouthpart, and generally depend on bacteriome-associated endosymbiotic microorganisms for provisioning of essential amino acids that are needed for protein synthesis but scarce in the plant sap (Moran et al., 2008; Douglas, 2009). The tree sap seeping out of the bark crevices is originally the same as the plant sap, but microbial proliferation and fermentation make it more nutrition-rich. In oak forests, for example, it is commonly found that microbe-fermented tree sap emits sweet alcoholic smell and attracts a huge number of flies, ants, wasps, moths, butterflies and beetles (Yoshimoto et al., 2005). Interestingly, however, the nosodendrid beetles are never found at such “crowded” tree sap sites. The tree sap of the non-oak trees that the nosodendrid beetles feed on (see Figures 1C,D,G,H, Table 1) seldom ferments in such a way, and attracts few other insects. We suspect that the nosodendrid beetles may adapt to the nutritionally poor tree sap that other insects do not utilize, where the endosymbiotic bacteria may play some roles. Chemical composition analysis of the tree sap in combination with metabolic information inferred from the endosymbiont genome may shed light on the physiological and nutritional mechanisms underlying the unique tree sap-feeding lifestyle of the nosodendrid beetles.

## Data Availability Statement

The datasets presented in this study can be found in online repositories. The names of the repository/repositories and accession number(s) can be found below: https://www.ddbj.nig.ac.jp/, LC567142, https://www.ddbj.nig.ac.jp/, LC567143, https://www.ddbj.nig.ac.jp/, LC567144, https://www.ddbj.nig.ac.jp/, LC567145.

## Author Contributions

BH conducted most of the experimental works, including sample collection, and histological, molecular and phylogenetic analyses. X-YM performed transmission electron microscopy. TF supervised the entire research project. BH and TF wrote the manuscript. All authors approved the final version of the manuscript.

## Conflict of Interest

The authors declare that the research was conducted in the absence of any commercial or financial relationships that could be construed as a potential conflict of interest.

## Supplementary Material

Supplementary Figure 1 Design of oligonucleotide probe, NosoFlav1263R, that specifically targets 16S rRNA of *N. coenosum* and *N. asiaticum*.

Supplementary Figure 2 Negative controls of FISH corresponding to Figure 4C. **(A)** Competitive suppression control with excess unlabeled probe. **(B)** No probe control. **(C)** RNase digestion control.

Supplementary Table 1 Number of nosodendrid samples subjected to different experiments.
