# Supplementary figures and images for: Bacteriome-Associated Endosymbiotic Bacteria of Nosodendron Tree Sap Beetles (Coleoptera: Nosodendridae)
Source: Front Microbiol. 2020 Oct 29;11:588841. doi: 10.3389/fmicb.2020.588841 (PMC7658545; doi:10.3389/fmicb.2020.588841)

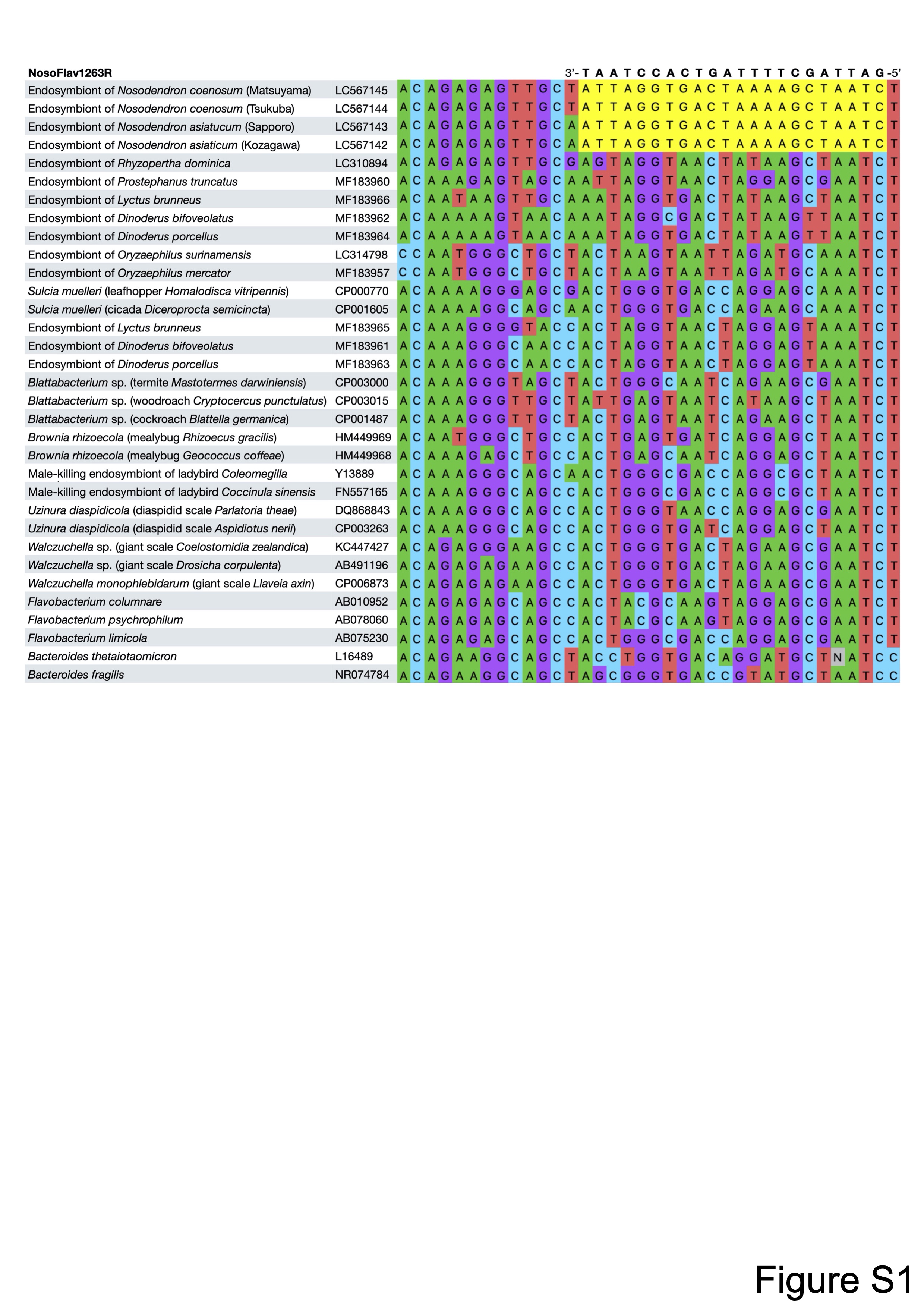

Supplement: Supplementary Figure 1 — Design of oligonucleotide probe, NosoFlav1263R, that specifically targets 16S rRNA of N. coenosum and N. asiaticum. [file Image_1.jpeg]

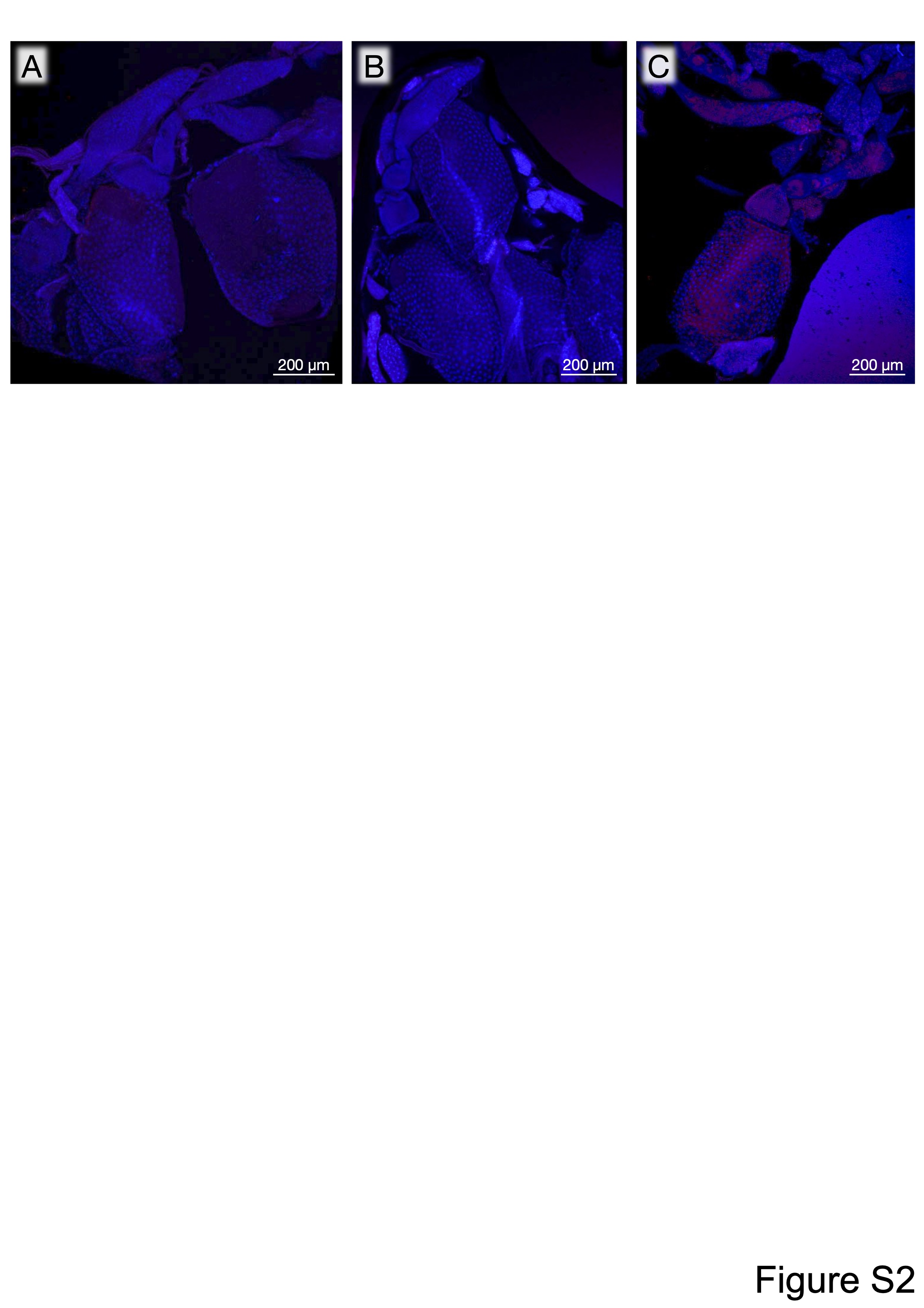

Supplement: Supplementary Figure 2 — Negative controls of FISH corresponding to Figure 4C. (A) Competitive suppression control with excess unlabeled probe. (B) No probe control. (C) RNase digestion control. [file Image_2.jpeg]
