# Supplementary material for: Bacteriome-Associated Endosymbiotic Bacteria of Nosodendron Tree Sap Beetles (Coleoptera: Nosodendridae)
Source: Front Microbiol. 2020 Oct 29;11:588841. doi: 10.3389/fmicb.2020.588841 (PMC7658545; doi:10.3389/fmicb.2020.588841)
Supplement: Supplementary Table 1 — Number of nosodendrid samples subjected to different experiments. [file Table_1.docx]

**Supplementary Table S1.** Number of nosodendrid samples subjected to different experiments.

| Insect species | *Nosodendron coenosum* | | *Nosodendron asiaticum* | |
| --- | --- | --- | --- | --- |
| Insect population | Tsukuba | Matsuyama | Kozagawa | Sapporo |
| Dissection and observation | F8 M6 L10 | F2 M2 L2 | A1 L2 | L2 |
| Symbiont 16S sequencing | L2 | L2 | L2* | L2* |
| Bacteriome FISH | F5 M2 L2 | - | - | L2 |
| Ovary FISH | F5 | - | - | - |
| Symbiont smear image | F1 | - | - | - |
| Transmission electron microscopy | L4 | - | - | - |
| Eggs for embryonic FISH | E12 | - | - | - |
| Total | F19 M8 L18 E12 | F2 M2 L4 | A1 L2 | L4 |

F, adult female; M, adult male; A, adult of unknown sex due to absence of gonads upon dissection; L, larva; E, egg. For example, F8 M6 L10 means that 8 adult females, 6 adult males and 10 larvae were examined.

^*^The same individuals as the samples used for dissection and observation.
